# Comparative Effect of Three Different Exercise Intensities in Combination with Diazoxide on Contraction Capacity and Oxidative Stress of Skeletal Muscle in Obese Rats

**DOI:** 10.3390/biology11091367

**Published:** 2022-09-17

**Authors:** Mariana Gómez-Barroso, Manuel A. Vargas-Vargas, Donovan J. Peña-Montes, Christian Cortés-Rojo, Alfredo Saavedra-Molina, Elizabeth Sánchez-Duarte, Alain R. Rodríguez-Orozco, Rocío Montoya-Pérez

**Affiliations:** 1 Instituto de Investigaciones Químico-Biológicas, Universidad Michoacana de San Nicolás de Hidalgo, Francisco J. Múgica S/N, Col. Felicitas del Río, Morelia 58030, Mexico; 2 Departamento de Ciencias Aplicadas al Trabajo, Universidad de Guanajuato, Campus León, Eugenio Garza Sada 572, Lomas del Campestre Sección 2, León 37150, Mexico; 3 Facultad de Ciencias Médicas y Biológicas “Dr. Ignacio Chavez”, Universidad Michoacana de San Nicolás de Hidalgo Av. Dr. Rafael Carrillo S/N Esq. Dr. Salvador González Herrejon, Bosque Cuauhtémoc, Morelia 58020, Mexico

**Keywords:** skeletal muscle, obesity, fatigue, oxidative stress, exercise, diazoxide

## Abstract

**Simple Summary:**

Obesity is a growing public health problem worldwide. It is a pathological state that degrades the proper functioning of skeletal muscle. Diazoxide treatment and exercise have been shown to generally improve muscle function. However, the effect that each of the different exercise intensities has when combined with diazoxide on the contraction capacity, resistance to fatigue and oxidative stress levels in rat skeletal muscle is unknown. Therefore, this work focused on analyzing which exercise intensity was more efficient in combination with diazoxide in improving muscle tissue and its metabolic capacities. The best results were obtained with low- and moderate-intensity exercise when combined with the drug. These results expected to open a window of time that allows the implementation of a constant and prolonged exercise protocol that completely reverses the harmful effects of obesity on muscle tissue and obesity itself.

**Abstract:**

Obesity is a chronic disease that impairs skeletal muscle function, affects the ability to contract, and promotes the development of fatigue. For this reason, the study of treatments that seek to reduce the harmful effects of obesity on muscle tissue has been deepened. Diazoxide treatment and various exercise protocols have been proposed to protect skeletal muscle against oxidative stress and its effects. However, the intensity and duration of exercise combined with diazoxide that would obtain the best results for improving skeletal muscle function in obese rats is unknown. To this end, this study evaluated the effects of three different exercise intensities combined with diazoxide on contraction capacity, resistance to fatigue, markers of oxidative stress, lipid peroxidation, ROS, and glutathione redox status of skeletal muscle. The results showed that treatments with diazoxide and exercise at different intensities improved muscle contraction capacity by reducing oxidative stress during obesity, with the best results being obtained with low-intensity exercise in combination with diazoxide. Therefore, these results suggest that diazoxide and low-intensity exercise improve muscle function during obesity by decreasing oxidative stress with the same efficiency as a moderate-intensity exercise protocol.

## 1. Introduction

Obesity is a chronic disease triggered by multiple factors, including disordered eating, lack of physical activity and sedentary lifestyle, or metabolic, hormonal, or genetic alterations [1,2]. It occurs when there is an imbalance between the ingested calories and those spent and is defined as the abnormal deposition of body fat detrimental to health [3]. It is classified as one of the main risk factors for human death, ranking fifth worldwide; it is a pathological state that has been identified as affecting skeletal muscle function, its contraction capacity, and fatigue resistance [2,4]; mentioned damage is characterized by an increase in metabolic and oxidative stress, and cell damage [5].

The increase in body fat during obesity is directly associated with the appearance of numerous metabolic and physiological processes, such as the modification in fiber type from slow to fast, a decrease in the number of fibers and their diameter; an accumulation of fat between the fibers, trigger insulin resistance, and establish a state of chronic inflammation [1,6,7], eventually affecting normal contractile function [8] and favoring the development of fatigue [9]. Also, a decrease in mitochondrial respiration and adenosine triphosphate (ATP) production occurs, as well as an increase in the production of reactive oxygen species (ROS) [10].

The increase in oxidative stress manifests itself in a very significant way in skeletal muscle during obesity, with the excessive increase in ROS levels, lipid disorders, and deregulation of antioxidant defense, which is directly reflected in the deterioration of muscle function [10,11].

In order to combat the harmful effects that obesity has on muscle tissue, pharmacological and non-pharmacological treatments have been studied in depth. For this purpose, treatment with diazoxide, a drug derived from benzothiadiazines, has been proposed [12]. Diazoxide, being an effective agonist of ATP-sensitive K+ channels (K_ATP_), inhibits insulin production and regulates the ability of skeletal muscle to take up glucose; it is a drug used in the treatment of dyslipidemia and obesity [13] since it decreases food consumption and body weight [14], improves fatty acid oxidation and decreases fat storage in the form of adipose tissue [13]. Studies such as that of Alemzadeh et al. in 2008 have shown that it improves the basal metabolic rate and fatty acid oxidation in obese Zucker rats, classifying it as an efficient anti-obesity treatment. Regarding its effects on the muscle tissue of obese models, it has been attributed to the improvement of muscle contraction and resistance to fatigue [12], as well as the decrease in metabolic and oxidative stress by reducing ROS levels and increasing antioxidant defense [13,15]. However, it is important to mention that recent studies have mentioned side effects with diazoxide treatment, mainly when used in human models, effects such as hypertrichosis, fluid retention, gastrointestinal reaction, edema, and neutropenia, so it is imperative to maintain monitoring and control while administering the drug.

In the case of non-pharmacological treatments for metabolic alterations associated with obesity, various exercise protocols have been suggested, with effective results obtained to prevent and counteract said alterations [16]. It has been observed that the application of exercise protocols promotes weight loss, prevents weight gain, and improves the lipid profile [17]. Improved glucose transport and insulin sensitivity are seen [18] and counteract oxidative stress [19,20].

Studies have identified that regular exercise is associated with numerous adaptations that improve the functioning of muscle tissue in obesity; it has been observed how fat stored between muscle fibers decreases [16], attenuates muscle inflammation [21], decreases oxidative tissue stress, and improves muscle contraction and resistance to fatigue [12].

However, the response obtained from the exercise will depend on the intensity, duration and the type of exercise applied [16]; the exercise can be classified in different intensities according to the VO2 max and the heart rate that is obtained [22]. The exercise protocol applied, will be determine from the physiological and metabolic response obtained [23]. In the case of obesity, there is contradictory information about which exercise intensity would be most optimal to treat the pathology and its direct effects on the functioning of muscle tissue. Recent studies show similar effects between high-intensity interval training and continuous training of moderate intensity, but without making it clear which one is recommended to improve the functioning of muscle tissue during obesity [2,16,23].

Therefore, it is essential to evaluate the effects that different exercise intensities have on the biochemical and physiological profiles of skeletal muscle during obesity, and to analyze their effectiveness when combined with other treatments that counteract the harmful effects of obesity at the systemic and tissue level, i.e., diazoxide.

To this end, the objective was to evaluate the effect of exercise at different intensities in combination with diazoxide on muscle contraction, resistance to fatigue, and oxidative stress of skeletal muscle in obese rats.

## 2. Materials and Methods

### 2.1. Experimental Groups

Male rats of the Wistar strain, weighing 300–350 g, were used at the beginning of the experiments. They were kept in acrylic cages under vivarium conditions at room temperature with periods of 12 h of light/12 h of darkness, with free access to food and water. The animals were randomly assigned to sixteen experimental groups, with n = 6 per group (see Table 1).

The diets were administered for 8 weeks; the standard diet for chow^®^ rodents had a content of 336 cal/100 g with a proportion of 28.507% protein, 13.496% fat, and 57.996% carbohydrate. The high-fat diet (surround chow^®^ and 50% fat [4] presented content of 649.25 cal/100 g with a proportion of 14.05% protein, 69.5% fat, and 21.4% fat (Mexican equivalent food system). Exercise protocols were executed for 8 weeks at three different exercise intensities. The exercise protocols were applied once a day, five days a week, with two rest days. A modified Centurfit ^®^ treadmill was used with an acrylic box divided into 8 lanes and equipped with movement sensors to produce air-based stimuli when required, and thus promote mobility of the rats [16] (see Table 2). Diazoxide was administered intraperitoneally at a concentration of 35 mg/kg for 14 days [24]. All treatments were applied under the Federal Standard for the Use and Care of Animals (NOM-062-ZOO-1999) issued by the Ministry of Agriculture of Mexico.

### 2.2. Monitoring of Body Weight and Blood Glucose Levels

During the treatment application, weight and glucose were monitored each week. A blood sample was taken by tail puncture (Accu-Chek^®^ Instant, Roche DC México S.A de C.V.); the visceral and perigonadal fat was obtained and quantified at the end of the treatment and after sacrifice.

### 2.3. Muscles Dissection

At the end of the treatment, the rats were subjected to a 12 h fast; after this period, they were sacrificed by decapitation. After sacrifice, the soleus and extensor digitorum longus (EDL) muscles of both hind limbs were dissected and harvested. A pair of muscles were held in Krebs-Ringer solution, and the isometric tension protocol was applied. Simultaneously, the other pair of muscles was stored at −80 °C to later homogenize for future biochemical tests [12].

### 2.4. Isometric Tension Measurements

Soleus and EDL muscles were kept submerged and perfused with Krebs-Ringer solution (118 mM NaCl, 4.75 mM KCl, 1.18 mM MgSO_4_, 24.8 mM NaHCO_3_, 1.18 mM KH_2_PO_4_, 10 mM glucose and CaCl_2_ 2.54 mM) and carbogen gas (95% O_2_ and 5% CO_2_). The muscle was cleaned and mounted as indicated in the methodology of Gómez-Barroso et al. 2020, in order to apply the fatigue induction protocol, which consisted of pulses of 100 V, 300 ms duration, and frequency of 45 Hz for soleus muscle and 50 Hz for EDL muscle. The stimulation was stopped once the fatigue and tension records were obtained, analyzed and quantified.

### 2.5. Reactive Oxygen Species Analysis 

ROS production was determined by reacting these molecules with the fluorescent probe 2′,7′-dichlorodihydrofluorescein diacetate (H2DCFDA). A total concentration of 0.5 mg/ml of protein was used, which was determined by the biuret method [25]. The protein was suspended in a buffer (HEPES 10 mM, KCl 100 mM, MgCl_2_ 3 mM, and KH_2_PO_4_ 3 mM, pH 7.4) and incubated with the H2DCFDA probe at a concentration of 12.5 µM for 15 min at 4 °C [26]. The basal fluorescence was obtained; after reading, the samples were returned to incubation at room temperature for 60 min, and the fluorescence was obtained again. Wavelengths for measurements consisted of 485/520 nm excitation/emission on a Shimadzu RF-5301PC spectrofluorophotometer. These results are expressed as arbitrary units (AU) per mg of protein.

### 2.6. Lipid Peroxidation Measurement

To determine lipid peroxidation, the levels of thiobarbituric acid reactive substances (TBARS) were analyzed [27]. 0.5 mg/ml of homogenate was used, suspended in 1 ml of phosphate buffer (50 mM KH_2_PO_4_, pH 7.6), and 2 ml of acid solution (15% trichloroacetic acid, 0.375%, and hydrochloric acid 0.25 M), the mixture was subjected to three cycles of agitation at low temperatures and then incubated for 15 min in boiling water. Finally, the samples were placed on ice for 5 minutes; after this time, the samples were centrifuged at 7500 rpm for 5 min. The absorbance of each sample was measured at a wavelength of 532 nm on a Shimadzu UV-2550 spectrophotometer using a molar extinction coefficient of 156 mM^−1^ cm^−1^ [28].

### 2.7. Determination of Glutathione Status Redox 

Muscle glutathione content was evaluated using 0.5 mg/ml sample protein. Samples were resuspended in 0.1 M phosphate buffer, 5.0 mM EDTA-Na2, 0.10% Triton-100X, and 0.6% 5-sulfosalicylic acid pH 7.5 and mixed vigorously.

They were then sonicated three times for 20 s on ice, followed by two freeze/thaw cycles, and centrifuged for 10 min at 8000 rpm. For total glutathione, 100 µL of the resulting supernatant was mixed and incubated for 30 seconds with 0.1 M phosphate buffer containing 5 mM EDTA-Na2, 0.1 mM 5,5-dithiobis-2-nitrobenzoic acid, 100 µM glutathione reductase enzyme, and 50 µM NADPH (reduced form). Changes in absorbance were recorded on a Shimadzu spectrometer (model UV-2550 UV VIS) at 412 nm at room temperature. Oxidized glutathione (GSSG) was assayed in 100 µL of supernatant previously incubated with 0.2% 4-vinylpyridine for 60 min. GSH was calculated by subtracting GSSG from total glutathione [28].

### 2.8. Data Analysis

Results were expressed as the mean ± standard error of independent experiments using samples from different animals. Statistical differences between groups were determined by one-way analysis of variance (ANOVA) and Tukey’s post hoc test. A *p* ≤ 0.05 was established. The analysis was performed with GraphPad Prism version 6.0 software (San Diego, CA, USA).

## 3. Results

### 3.1. Effect of Different Exercise Intensities and Diazoxide on Weight, Glucose, and Visceral and Perigonadal Fat in Obese Rats

The effect of different exercise intensities, diazoxide, and their respective combinations on body weight, fasting serum glucose levels at the end of each treatment and after sacrifice, and the weight of visceral and perigonadal fat was quantified. After applying the various treatments for 8 weeks, phenotypic and biochemical were observed. Table 3 shows these values. As expected, in the obese group, body weight, plasma glucose levels, and visceral and perigonadal fat increased; however, in the treated obese rats, a decrease in these parameters was observed, and such effect was observed with all the protocols applied. The best results were obtained with low-intensity and moderate-intensity exercise combined with the drug, registering a 30% (*p* = 0.024, 0.018) reduction in body weight, between 61–67.5% (*p* = 0.022, 0.016) in plasma glucose levels and 40% (*p* = 0.042, 0.039) in visceral and perigonadal fat weight vs. the obese group without treatment.

### 3.2. Different Exercise Intensities and Diazoxide Enhance Contraction and Promote Fatigue Resistance of Skeletal Muscle in Obese Rats

**Figure 1:**
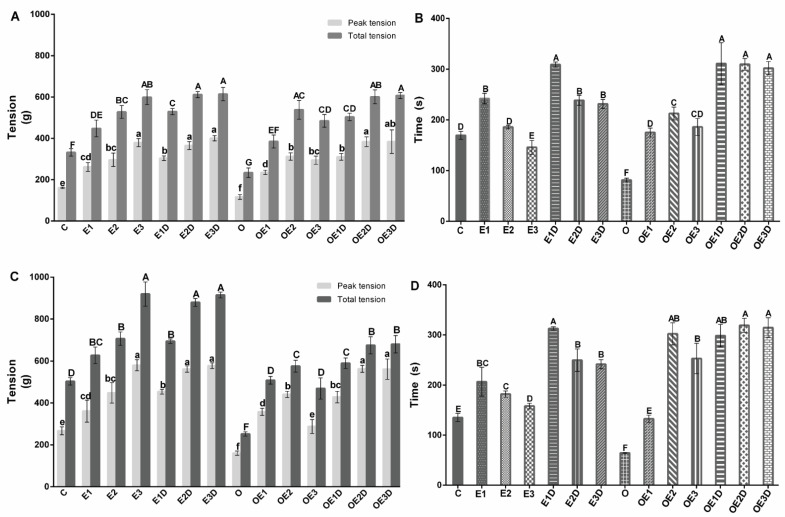
Effect of different intensities of exercise and diazoxide on muscle contraction and resistance to fatigue. (**A**) Maximum tension and total tension of the soleus muscle, (**B**) Endurance time to fatigue of the soleus muscle, (**C**) Maximum and total tension of the EDL muscle, (**D**) Endurance time to fatigue of the EDL muscle. C: control; D: diazoxide; E1: low-intensity exercise; E2: moderate-intensity exercise; E3: high-intensity exercise; E1D: low-intensity diazoxide exercise; E2D: exercise with moderate intensity diazoxide; E3D: exercise with high-intensity diazoxide; O: obese; OD: obese diazoxide; OE1: low-intensity exercise for obese; OE2: obese athlete of moderate-intensity; OE3: obesity due to high-intensity exercise; OE1D: low-intensity exercise diazoxide in obese; OE2D: moderate-intensity exercise for obese; OE3D: high-intensity diazoxide exercise for obese. Data are represented as mean ± standard error. Different letters indicate statistically significant differences between groups (*p* < 0.05) 1-way ANOVA, Tukey’s posthoc test, n = 6.

To explore the effect of treatment with diazoxide, the different exercise intensities, the combination of both the maximum and total tension, and the time of resistance to fatigue, recording of the isometric tension in soleus muscle and EDL of the different groups was performed. Figure 1 shows the soleus muscle’s maximum and total tension and endurance time (Figure 1A,B) and EDL (Figure 1C,D). The graphs show how muscle contraction and fatigue resistance are affected by obesity in both types of muscle, presenting significant decreases compared to the control group (*p* = 0.036, 0.017, 0.0058, 0.041, 0.011, 0.0078). The treatment with the different exercise protocols, combined with diazoxide, increased the force of contraction and the time of resistance to fatigue in both types of muscle during obesity (*p* < 0.05), observing the best results with moderate-intensity exercise in combination with diazoxide and presenting significant increases of than 60% (*p* = 0.016, 0.013, 0.0058, 0.0096, 0.0088, 0.0023) vs. the obese group without treatment.

### 3.3. Different Intensities of Exercise and Diazoxide Decrease Oxidative Stress and Improve Antioxidant Defense in Skeletal Muscle of Obese Rats

**Figure 2:**
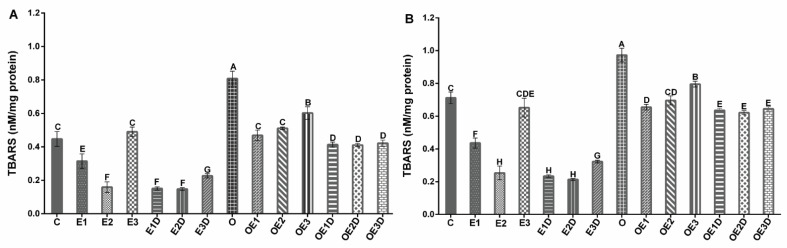
Effect of different exercise intensities and diazoxide on ROS of skeletal muscle in obese rats. (**A**) Soleus muscle (**B**) EDL muscle. C: control; D: diazoxide; E1: low-intensity exercise; E2: moderate-intensity exercise; E3: high-intensity exercise; E1D: low-intensity diazoxide exercise; E2D: exercise with moderate intensity diazoxide; E3D: exercise with high-intensity diazoxide; O: obese; OD: obese diazoxide; OE1: low-intensity exercise for obese; OE2: obese athlete of moderate-intensity; OE3: obesity due to high-intensity exercise; OE1D: low-intensity exercise diazoxide in obese; OE2D: moderate-intensity exercise for obese; OE3D: high-intensity diazoxide exercise for obese. Data are represented as mean ± standard error. Different letters indicate statistically significant differences between groups (*p* < 0.05) 1-way ANOVA, Tukey’s posthoc test, n = 6.

**Figure 3:**
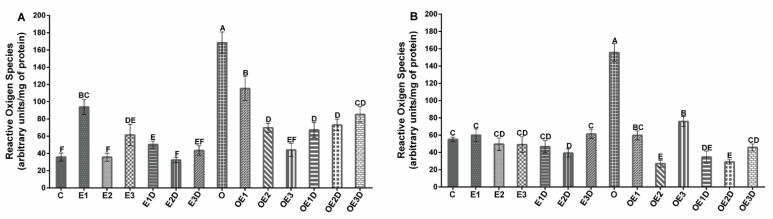
Effect of different exercise intensities and diazoxide on lipid peroxidation of skeletal muscle in obese rats. (**A**) Soleus muscle (**B**) EDL muscle. C: control; D: diazoxide; E1: low-intensity exercise; E2: moderate-intensity exercise; E3: high-intensity exercise; E1D: low-intensity diazoxide exercise; E2D: exercise with moderate intensity diazoxide; E3D: exercise with high-intensity diazoxide; O: obese; OD: obese diazoxide; OE1: low-intensity exercise for obese; OE2: obese athlete of moderate-intensity; OE3: obesity due to high-intensity exercise; OE1D: low-intensity exercise diazoxide in obese; OE2D: moderate-intensity exercise for obese; OE3D: high-intensity diazoxide exercise for obese. Data are represented as mean ± standard error. Different letters indicate statistically significant differences between groups (*p* < 0.05) 1-way ANOVA, Tukey’s posthoc test, n = 6.

To evaluate the oxidative stress in skeletal muscle of obese rats, we determined the levels of TBARS as an indicator of lipid peroxidation and the levels of ROS. Figure 2 shows the TBARS levels for soleus muscle (2A) and EDL muscle (2B), and Figure 3 shows the ROS levels for soleus muscle (3A) and EDL muscle (3B). The graphs show an increase of over 40% (*p* = 0.029, 0.016) in lipid peroxidation levels and 300% (*p* = 0.0067, 0.0074) in ROS levels during obesity. Notwithstanding the treatments applied, the three different exercise protocols and their respective combinations with diazoxide showed beneficial effects on these parameters, with slight variations between them. They present a decrease in both TBARS and ROS levels in both muscles; the low-intensity and moderate-intensity exercise protocols in combination with diazoxide showed the most significant results, presenting reductions greater than 100% (*p* < 0.05) vs. the obese group without treatment.

**Figure 4:**
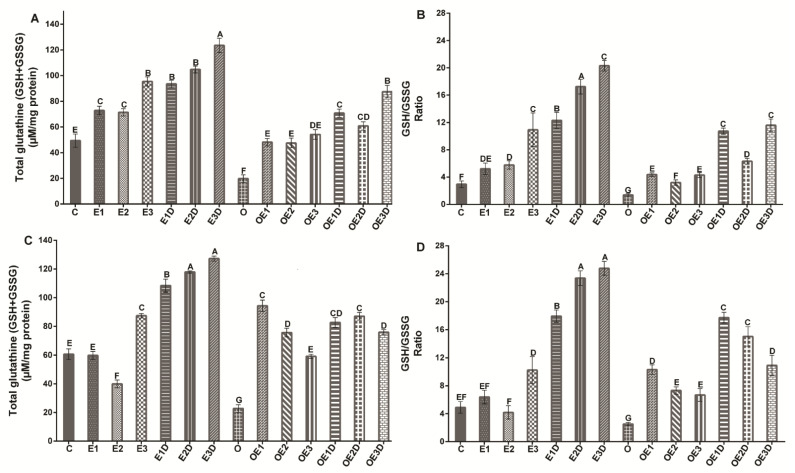
Effect of different exercise intensities and diazoxide on the glutathione redox state of skeletal muscle in obese rats. (**A**) Total glutathione soleus muscle, (**B**) Ratio soleus muscle, (**C**) Total glutathione EDL muscle, (**D**) Ratio EDL muscle, C: control; D: diazoxide; E1: low-intensity exercise; E2: moderate-intensity exercise; E3: high-intensity exercise; E1D: low-intensity diazoxide exercise; E2D: exercise with moderate intensity diazoxide; E3D: exercise with high-intensity diazoxide; O: obese; OD: obese diazoxide; OE1: low-intensity exercise for obesity; OE2: obese moderate-intensity exerciser; OE3: obesity due to high-intensity exercise; OE1D: low-intensity exercise diazoxide in obesity; OE2D: moderate-intensity exercise for obesity; OE3D: high-intensity diazoxide exercise for obese. Data are represented as mean ± standard error. Different letters indicate statistically significant differences between groups (*p*< 0.05) 1-way ANOVA, Tukey’s posthoc test, n = 6.

To determine if the lower levels of lipid peroxidation and ROS caused by the different exercise and diazoxide protocols in the muscles of obese rats were directly proportional to the changes in the redox state, the glutathione antioxidant defense system (Figure 4) was analyzed in soleus muscle (Figure 4A,B) and EDL muscle (Figure 4C,D). The redox status of glutathione changed considerably in the muscles of obese rats compared to the muscles of control rats, with a decrease in both total glutathione and the ratio of reduced glutathione/oxidized glutathione (GSH/GSSG). A decrease in total glutathione of 52.3% (*p* = 0.021) was observed, and a decrease in the GSH/GSSG ratio of 71.3% % (*p* = 0.012) in soleus muscle and 66.88%(*p* = 0.018) and 54.9% (*p* = 0.036) in EDL muscle. The various treatments applied in the groups of obese rats improved both total glutathione and the GSH/GSSG ratio. However, the low-intensity exercise treatment in combination with diazoxide presented the best results in both muscles, with significant increases of more than 200% in total glutathione and the GSH/GSSG (*p* < 0.05). These results show how diazoxide potentiates the effects of low-intensity exercise, increasing antioxidant defense and, therefore, decreasing oxidative stress.

## 4. Discussion

The anatomical and functional alterations of skeletal muscle during obesity are complex: this pathology causes muscle atrophy [29], metabolic alterations, and oxidative stress, which leads to a diminution in the capacity of muscle contraction and fatigue resistance [3,9]. Eventually, this alteration will have adverse effects on the entire system, altering the metabolism and triggering other pathologies, such as diabetes, hypertension and even some types of cancer [5,19,30]. 

In order to counteract the harmful effects of obesity on skeletal muscle tissue, this study compares the effects of three different exercise protocols, which differed from each other in intensity and duration [16], together with the application of diazoxide, a drug derived from benzothiadiazines, which has substantial effects on metabolism during obesity and on the functioning of muscle tissue in general [12,14].

This experimental series was applied to analyze the muscle response to these treatments in the context of diet-induced obesity. The study sought to determine which exercise protocol was most efficient in combination with diazoxide to improve the functioning of muscle tissue and, therefore, its metabolic capacities, thereby opening a window of time that allows the implementation of a constant and prolonged exercise protocol that fully reverses the effects of obesity on skeletal muscle, and eventually obesity itself.

Previous studies have shown that obesity induced by a high-fat diet alters many biochemical and physiological parameters, increases body weight and plasma glucose, triggers insulin resistance, and alters the lipid profile in Wistar rats [1,11,14,30]. We have verified this with our results (Table 3). We observed how weight, glucose, and fat are altered during obesity concerning control. These altered parameters are indicators of alterations in muscle cell metabolism, which is eventually associated with obesity, deterioration of muscle strength, and resistance to fatigue [31]. However, in this study, it was perceived that the treatment with the different exercise protocols and their respective combinations with diazoxide decreased body weight, plasma glucose, and visceral and perigonadal fat during obesity (Table 3). The mentioned effects are associated with the improvement of the basal metabolic rate, the increase in sensitivity to insulin and glucose transport and suppression of hyperinsulinemia, and the increase in the capacity of oxidation of fats [3,14,18,21].

In healthy models, the literature has reported the best effects for moderate-intensity exercise [2,5,32], which is consistent with our results (Table 3). During obesity, it was observed that moderate-intensity exercise shows the best effects on these parameters; however, when combining exercise with diazoxide, this effect was potentiated, showing the best results with low- and moderate-intensity exercise when combined with the drug (Table 3). The results obtained with low-intensity exercise in combination with diazoxide should be highlighted since they are similar to those obtained with moderate-intensity exercise when combined with the drug, which is beneficial and outstanding because the low-intensity protocol is easier to execute.

As we have already mentioned, the alteration of the biochemical and physiological parameters produced by obesity triggers dysfunction of the skeletal muscle tissue [9]. In this experimental series, a decrease in muscular contraction and fatigue resistance was observed in soleus skeletal muscle and EDL of fat rats (Figure 1). The results show off how obesity affects the contractility of both muscles, which is associated with a rise in intramuscular fat [1,9], change in muscle fiber type, metabolic alterations, and an increase in oxidative stress during exercise [2,3,10,33].

However, prior studies have shown that diazoxide has positive effects by preventing and even reversing metabolic disorders that are related to obesity, such as improving insulin sensitivity, which has been associated with a rectification in glucose transport and lipid metabolism [13,14,15], favoring muscle function. In previous works of our group, the beneficial action of diazoxide treatment on the muscle function of obese rats was confirmed [12]. Improving the muscle metabolic capacities and promoting the opening of KATP channels by diazoxide generates a rise in cellular respiration through better functioning of the electron transport chain (ETC) and, therefore, an improvement in the production of ATP [34,35]. This has a direct effect on improving muscle contraction and reducing muscle fatigue [36,37].

Similarly, exercise has a significant effect on promoting adaptations that improve the metabolic capacities of muscle and its functioning [2,17,38], among which are the reduction in intramuscular fat resulting from an increase in skeletal muscle metabolism [2,5,20], regulation of signaling pathways involved in the optimization of insulin sensitivity and glucose transportation, and the reduction in the lipid outline and stress markers [3,19]. Our results show how the three exercise protocols improve the contraction capacity and resistance to fatigue in the healthy model’s two types of muscle. However, in the case of muscle contraction, high-intensity exercise presented the best results, while, in the case of fatigue resistance, we observed that it was low-intensity exercise that presented them (Figure 2). This is dependent on the type of exercise applied, the muscular adaptations promoted, and the physiological and metabolic response that is obtained [16,22]. At the time of implementing diazoxide into the exercise protocols, it was possible to see how the beneficial effects of exercise were potentiated by presenting with greater capacity for contraction and resistance to fatigue compared to its individual application.

In the pathology case, we observed how all the protocols improved the contraction capacity and the fatigue resistance in both types of muscle, which indicates the plasticity capacity of the muscular tissue to adapt to the exercise type [20,38]. Better results were seeing when moderate-intensity exercise in combination with diazoxide was applied (Figure 2), this is consistent with what had been previously reported in our previous study [12]. However, the effect of low-intensity exercise in combination with diazoxide should be highlighted since, when combining this type of exercise with the drug, the effect obtained significantly exceeded, the effects obtained with moderate-intensity exercise applied alone. These results are very relevant since they show that a type of exercise that is easier to apply, such as low-intensity exercise, when combined with the drug equals the results that can be obtained with moderate-intensity exercise.

Obesity increases oxidative stress, eventually damaging muscle tissue function [11,39]. For the proper functioning of skeletal muscle certain concentrations of ROS are needed which are generated both under conditions of rest and during contractile activity [6,40,41]. However, excessive ROS concentrations produced in muscle under certain circumstances cause increased oxidative stress and eventually compromise muscle function [41,42]. During obesity, the increase in free fatty acids and fat storage in skeletal muscle has been related to the development of insulin resistance and a chronic inflammatory state involved in increased ROS levels, lipid peroxidation, and decreased antioxidant defense [6,10].

In this work, different oxidative stress markers were evaluated to analyze their presence during obesity and determine the effects of the different treatments on these markers. For this, the levels of ROS (Figure 2), the levels of TBARS (Figure 3), and the redox state of glutathione (Figure 4) were analyzed.

ROS is one of the primary markers of oxidative stress, the excessive accumulation of these molecules is associated with structural and functional damage to cells. During obesity, there is excessive production of ROS [19,23]. There is a relationship between the amount of ROS produced at the systemic level and in the muscle, and the reduction in muscle contractility and appearance of fatigue [43]. In our results, we can observe how during obesity, there is an overproduction of ROS in muscle tissue (Figure 2). During the pathology, it can have various sources of origin, including hyperglycemia, excess free fatty acids, cytokine production, and excess tissue that must be oxygenated [19]. The application of regular exercise protocols decreases ROS levels [17]. In the case of obesity, it can be seen how the different protocols applied decreased ROS levels, with low- and moderate-intensity exercise presenting the best results. In combination with diazoxide (Figure 2), this effect of exercise had been observed in other studies, mainly when applied at moderate intensity [10,44,45]; however, our results show that similar results are obtained with low-intensity exercise when combined with the drug.

One of the main effects of the increase in ROS is the damage to muscle cell biomolecules, such as proteins, lipids, and nucleic acids [46]. Therefore, in this study the levels of TBARS, an indicator of peroxidation, lipid and oxidative stress were quantified. When there is an increase in ROS production, as in the case of obesity, damaging effects can be produced on the membrane’s phospholipids, and as a result, malondialdehyde (MDA) is formed, which is toxic to cells [19,47]. This has been reported to alter the functioning of the ETC, affecting its activity and therefore the production of ATP, which contributes to the fatigue and stress of muscle cells [12,48]. In our results, we can observe in the healthy models for both types of muscle that moderate-intensity exercise presented the best result by decreasing TBARS levels (Figure 3), consistent with what has been reported in the literature where said effect is attributed to this type of exercise [19,49].

On the other hand, obesity increases the levels of lipid peroxidation in muscle tissue; this is associated with the increase in ROS, the increase in adipose tissue and its lipolysis, triggering of lipid peroxidation and causing structural and functional damage of the muscle cell, which interferes with its ability to contract and resist fatigue [17]. However, when applying the different treatments during obesity in both muscles, a decrease in lipid peroxidation was observed with the three exercise protocols showing the best results in the combination with diazoxide (no statistical differences) (Figure 3), showing how the drug potentiates the effects of exercise. It should be mentioned that this effect is more compatible when combined with low-intensity exercise. The decrease in lipid peroxidation during obesity is associated with the improvement in metabolic and functional capacities of the muscle and, above all, its capacity for contraction and fatigue resistance (Figure 1) [23].

Antioxidant systems in the cells protect them against oxidative stress. Glutathione is included in this system, whose synthesis can be altered by exercise, diet, and age [17,43]. In this study, it was possible to observe how oxidative stress increased as a consequence of obesity-induced skeletal muscle atrophy and vice versa and how, during this pathology, the antioxidant response is insufficient, which promotes the overproduction of ROS and the oxidation of lipids in the cells of the muscle fibers [6,23,47]. Our results showed that total glutathione and the GSH/GSSG ratio of the soleus and EDL muscle (Figure 4) were decreased during obesity, suggesting that obesity interferes with the synthesis of this antioxidant, possibly because the ROS that are overproduced during this disease are related in the activation of signaling pathways that adversely affect glutathione synthesis such as NF-Kb, Sp1, E [11,23,50].

However, the different treatments applied during obesity improve the levels and ratio of glutathione in soleus muscle and EDL, with low-intensity exercise combined with diazoxide presenting the best results (Figure 4). By increasing the antioxidant defense, the oxidative stress caused by obesity is combated, and the harmful effects caused by it can be reversed; that is, it improves muscle function, increases the contraction capacity, and promotes resistance to obesity fatigue [19,51].

In healthy models, it can be seen how high-intensity exercise presented the most significant increase in antioxidant defense (Figure 4), proving how high-intensity exercise attenuates oxidative damage by increasing glutathione levels [23]. However, when combining the protocols with diazoxide, the effects of all of them were potentiated, presenting in this model very similar results between moderate- and high-intensity exercise.

## 5. Conclusions

Diazoxide potentiates the effects of exercise, regardless of its intensity and duration. The best results during obesity were obtained by combining the drug with low-intensity exercise, resulting in the treatment with the best effects on muscle contraction and fatigue resistance by reducing ROS levels, lipid peroxidation, and enhancing the redox state of glutathione. In healthy models, we rectified that the best effects occur when a moderate intensity protocol is applied and how diazoxide also potentiates these effects.

## Figures and Tables

**Table 1:** Experimental groups.

Groups	Diet	Diazoxide 35 mg/kg	Exercise
Control (C)	Standard rodent chow^®^	no	no
Diazoxide (D)	Standard rodent chow^®^	yes	no
Low-intensity exercise (E1)	Standard rodent chow^®^	no	yes
medium intensity exercise (E2)	Standard rodent chow^®^	no	yes
High-intensity exercise (E3)	Standard rodent chow^®^	no	yes
Low-intensity exercise diazoxide (E1D)	Standard rodent chow^®^	yes	yes
Medium intensity exercise diazoxide (E2D)	Standard rodent chow^®^	yes	yes
High-intensity exercise diazoxide (E3D)	Standard rodent chow^®^	yes	yes
Obese (O)	High-fat diet	no	no
Obese diazoxide (OD)	High-fat diet	yes	no
Obese low-intensity exercise (OE1)	High-fat diet	no	yes
Obese medium intensity exercise di (OE2)	High-fat diet	no	yes
Obese low-intensity exercise diazoxide (OE3)	High-fat diet	no	yes
Obese low-intensity exercise diazoxide (OE1D)	High-fat diet	yes	yes
Obese medium-intensity exercise diazoxide (OE2D)	High-fat diet	yes	yes
Obese low-intensity exercise diazoxide (OE3D)	High-fat diet	yes	yes

The diets were administered for 8 weeks, while the exercise protocols were applied individually at three different exercise intensities (low, medium, and high) for 8 weeks. Diazoxide was administered intraperitoneally for 14 days at a 35 mg/kg dose. Finally, combinations of treatments were performed, respectively.

**Table 2:** Exercise protocol.

Groups	E1	E2	E3
Week 1	10 m/min/10 min	10 m/min/10 min	10 m/min/15 min
Week 2	10 m/min/10 min	10 m/min/15 min	10 m/min/20 min
Week 3	10 m/min/10 min	10 m/min/15 min 17 m/min/5 min	17 m/min/15 min
Week 4	10 m/min/15 min	10 m/min/15 min 17 m/min/10 min	17 m/min/20 min
Week 5	10 m/min/15 min	10 m/min/15 min 17 m/min/10 min 22 m/min/5 min	17 m/min/10 min 22 m/min/10 min
Week 6	10 m/min/20 min	10 m/min/15 min 17 m/min/10 min 22 m/min/5 min	17 m/min/10 min 22 m/min/10 min
Week 7	10 m/min/15 min 17 m/min/5 min	10 m/min/20 min 17 m/min/15 min 22 m/min/5 min	17 m/min/5 min 22 m/min/20 min
Week 8	10 m/min/15 min 17 m/min/10 min	10 m/min/20 min 17 m/min/15 min 22 m/min/5 min	17 m/min/10 min 22 m/min/25 min

Exercise protocol per week for groups: E1; low-intensity exercise, E2; moderate intensity exercise, E3; high-intensity exercise. The speed is displayed in meters per minute by the time during which this speed was applied.

**Table 3:** Body weight and visceral and perigonadal fat, plasma glucose levels.

Groups	Bodyweight (g)	Glucose (mg/dL)	Viceral and Perigonadal Fat (g)
C	408.245 ± 18.18 ^D^	73.83 ± 6.04 ^E^	11.07 ± 6.04 ^D^
D	413.5 ± 15.23 ^D^	86.66 ± 3.66 ^C^	9.84 ± 2.84 ^D^
E1	425.33 ± 39.62 ^CD^	77 ± 5.09 ^EF^	6.2 ± 2.24 ^E^
E2	435.8 ± 15.75 ^CD^	69.2 ± 4.08 ^G^	6.28 ± 3.43 ^E^
E3	417.5 ± 11.18 ^D^	69 ± 2.73 ^G^	5.8 ± 2.49 ^E^
E1D	588.83 ± 32.86 ^A^	111 ± 4.09 ^A^	43.32 ± 4.38 ^A^
E2D	483.8 ± 23.92 ^B^	73.83 ± 2.22 ^E^	18.47 ± 3.33 ^C^
E3D	497 ± 16.68 ^B^	82.85 ± 6.25 ^CD^	21.39 ± 4.60 ^C^
O	455.14 ± 40.71 ^C^	85.71 ± 4.75 ^C^	21.61 ± 3.79 ^C^
OD	515 ± 67.32 ^B^	105.16 ± 6.55 ^B^	30.88 ± 8.14 ^B^
OE1	441.16 ± 30.43 ^C^	74.5 ± 2.88 ^E^	19.16 ± 4.84 ^C^
OE2	441.75 ± 19.55 ^C^	68.83 ± 3.54 ^G^	20.11 ± 5.94 ^C^
OE3	461.13 ± 17.03 ^C^	79 ± 2.58D ^F^	11.25 ± 3.61 ^D^
OE1D	406.4 ± 10.23 ^D^	81.8 ± 2.38 ^D^	11.85 ± 2.17 ^D^
OE2D	404.25 ± 7.45 ^D^	70.5 ± 4.66 ^G^	10.30 ± 3.12 ^D^
OE3D	402.75 ± 10.56 ^D^	73.6 ± 3.43 ^EG^	6.62 ± 1.23 ^E^

Effect of different exercise intensities and diazoxide on weight, glucose, and visceral and perigonadal fat in obese rats: C: control; D: diazoxide; E1: low-intensity exercise; E2: moderate-intensity exercise; E3: high-intensity exercise; E1D: low-intensity diazoxide exercise; E2D: moderate-intensity diazoxide exercise; E3D: high-intensity diazoxide exercise O: obese; OD: obese diazoxide; OE1: obese low-intensity exercise; OE2: obese moderate-intensity exercise; OE3: high-intensity exercise obese; OE1D: obese low-intensity exercise diazoxide; OE2D: obese exercise moderate intensity; OE3D: obese high-intensity diazoxide exercise. Data are represented as mean ± standard error. Different letters indicate statistically significant differences between groups (*p* < 0.05) 1-way ANOVA, Tukey’s posthoc test, n = 6.

## Data Availability

This article finds all the data analyzed and obtained.
